# Diversity of allergen exposure: Implications for the efficacy of environmental control

**DOI:** 10.1016/S1808-8694(15)30796-5

**Published:** 2015-10-19

**Authors:** Gesmar Rodrigues Silva Segundo, Mônica Camargo Sopelete, Sílvia Azevedo Terra, Fernando Lourenço Pereira, Caroline Morais Justino, Deise Aparecida de Oliveira Silva, Ernesto Akio Taketomi

**Affiliations:** 1 Master's degree, doctoral student in immunology and applied parasitology, Universidade Federal de Uberlandia; 2 Doctorate, post-doctoral student in allergy and clinical immunology, Universidade Federal de Uberlandia; 3 Master's degree, assistant professor of veterinary immunology, Ituverava, SP; 4 Master's degree, doctoral student in applied immunology, FMRP/USP; 5 Master's degree, basic education teacher, Santos, SP; 6 Doctorate, researcher and supervisor of the Allergy and Clinical Immunology Laboratory, Universidade Federal de Uberlandia; 7 Doctorate / post-doctorate, full professor of immunology and head of the Allergy and Clinical Immunology Laboratory, Universidade Federal de Uberlandia. Universidade Federal de Uberlandia

**Keywords:** aeroallergen, allergen, environment, asthma, allergen exposure, allergic rhinitis

## Abstract

The prevalence of allergic diseases such as asthma, rhinitis, allergic conjunctivitis and atopic dermatitis has increased in the last decades. The relationship between allergen exposure, atopic sensitization and development of allergic diseases is widely described in the literature.

**Aim:**

To evaluate measures for reducing allergen exposure as part of the treatment of allergic diseases.

**Methods:**

An analysis was made of previous studies on allergen exposure done with a similar methodology in the central region of Brazil; the study included homes, hotels, cinemas, cars, taxis, buses and scholar transportation.

**Results:**

High levels of Der p 1 and Der f 1 mite allergens were found in a large proportion of the sample in most of the environments included in those studies; there were higher levels of pet allergens in cars and school transportation vehicles.

**Conclusion:**

The diversity of allergen exposure demonstrates the need for education about allergic diseases for patients and their families, as well as measures of reducing allergens in homes. This should be part of a global strategy of the management of allergic diseases, given that individuals live in society, not only in their houses.

## INTRODUCTION

Atopy is the term for individuals whose immune system produces IgE antibodies in response to specific antigenic stimuli. Allergy is the term for an altered state resulting from an immunological process set in motion by a substance or allergen that is inert^1^ or that induces a non-IgE mediated antibody response in normal individuals. The majority of allergic diseases is caused by the type I (immediate) hypersensitivity reaction, which is an IgE-mediated abnormal immune response.

The prevalence of allergic diseases such as asthma, rhinitis, allergic conjunctivitis and atopic dermatitis has increased in the past few decades.^2^ ISAAC data recently described in Brazil show that the prevalence of asthma may reach 28.2%, depending on which center is studied.^3^ Another Brazilian multicentric study revealed a significant difference in air allergen sensitization between allergic individuals and healthy controls, demonstrating the relation between atopy and these conditions.^4^ Other authors have shown that there is atopic sensitization in over 40% of young adults in the USA.^5^

Kern (1921) first described house dust as an allergen; his report showed that many asthma and rhinitis patients developed skin erythema and edema due to dust extracts from their own houses.^6^ Various sources of house dust allergens had been described by 1960, including animal dander, insects and fungi. In 1967, a mite in dust (Dermatophagoides pteronyssinus) was shown to cause a skin reaction; it has since been considered as the main house dust allergenic agent in the Netherlands, and subsequently in other countries such as the United Kingdom, Australia, Japan and Brazil.^7^

House dust allergens were purified during the following decades; the cat (Felis domesticus) allergen – Fel d 1 – was the first to be described, in 1974.^8^ In 1980, the first mite allergen, Der p 1 (derived from Dermatophagoides pteronyssinus), was isolated and characterized.^9^

Mite detection in house dust was done in the past by microscopy and acarid count per square centimeter; quantification of allergens in different sites, however, was not possible at that time. After the development of monoclonal antibodies (a highly sensitive and specific tool for identifying and quantifying antigens), many authors have standardized immunoenzymatic tests for investigating allergen levels in house dust and environmental extracts, which have underlined environmental prevention measures.^10^

The relation between exposure, atopic sensitization and the development of allergic disease has been widely described in the literature. Basic evidence for a cause-effect relation between allergen exposure and sensitization arose from studies showing that children in places with no mites in house dust (Northern Sweden, for instance) were not sensitized to mite allergens. Likewise, children brought up with little or no exposure to cockroaches (in places such as New Zealand, Sweden or Delaware-USA) were not sensitized to cockroach allergens.^11^

Prevalence, allergen exposure, and sensitization studies of asthmatic children and adults have suggested that exposure to over 2μg of Der 1 allergens per gram of dust is a risk factor in genetically predisposed individuals. The allergen risk factor for animal dander (Can f 1, Fel d 1) is exposure to over 1μg/g dust. These values are currently believed to vary significantly, as allergic diseases have been considered as multifactorial conditions that depend on the interaction between many factors, such as inheritance, diet, permanence time in closed environments, age of contact and others.^12^, ^13^, ^14^, ^15^, ^16^ As allergen contact is one of the factors causing inflammation in allergic diseases (especially in asthma and allergic rhinitis), environment control has become a mainstay in the treatment of these conditions, and has been recommended by national and international consensuses. Various published papers had shown that there are variable responses to allergen control measures; the study design and the response assessments, however, varied among these papers, which has generated controversies among physicians.^17^, ^18^, ^19^, ^20^, ^21^, ^22^, ^23^, ^24^, ^25^, ^26^, ^27^, ^28^

The purpose of this paper was to discuss the difficulty of controlling allergen exposure and to review various studies in different environments within which individuals spend their days.

## METHOD

This review was composed of published studies about allergen exposure undertaken in the central region of Brazil. The climate (humidity and temperature) was similar in all studies, as were the allergen dosing techniques in various closed environments. Sampling included 124 houses, 5 movie theater rooms, 20 hotels, 60 private vehicles, 120 interstate passenger buses, 60 taxis and 60 school children transport vans.^29^, ^30^, ^31^, ^32^, ^33^, ^34^, ^35^, ^36^

After obtaining approval from participants, dust samples were collected in each site by a portable aspirator onto which a paper filter had been adapted to retain the dust. Dust was then placed in a plastic bag and identified; storage was at 4°C before allergens were extracted. Dust samples were sieved using a standard 0.3 mm pore sieve, Series ASTM (USA), onto a Petri dish and then transferred to test tubes. Allergen fractions were extracted from 100 mg of dust from each sample plus 2 ml of a borate buffered saline solution (BBS), at 5 mM, pH 8.0, at 4°C, rotatory shaking, during 18 hours. Samples were then centrifuged at 10,000 g for ten minutes; the supernatant was stored at −l20°C for future analysis of its allergen content.

The enzyme-linked immunosorbent assay (ELISA) was used for detecting the Der p 1, Der f 1, Can f 1 and Fel d 1 allergens as described by Luczynska et al.^37^ modified by Sopelete et al.^28^ The following monoclonal antibodies (mAb) for capture were used: anti-Der p 1 (clone 5H8), anti-Der f 1 (clone 6A8), anti-Fel d 1 (clone 6F9), and anti-Can f 1 (clone 6E9); the concentration was 10 mg/ml in a carbonate-bicarbonate buffer at 0.06M and pH 9.6. Detection was done using biotinilated mAb, as follows: anti-Der p 1 and anti-Der f 1 (4C1), anti-Fel d 1 (3F4C4) and polyclonal rabbit serum anti-Can f 1 at 1:500. After incubation with streptavidine-peroxidase (Sigma Chemical Co., USA), the test tube was developed by adding an enzymatic substrate (0.01 M ABTS and 0.03% H2O2); reading was done at 405nm. Reference standards containing known levels of each allergen were included into each plate in double to obtain curves for eleven double serial dilutions starting at 250 ng/ml for Der p 1 and Der f 1, 500 ng/ml for Can f 1, and 80ng/ml for Fel d 1. Results were expressed in μg/g of dust.

The Graph Pad Prism version 3.0 (Graph Pad Software, Inc.) software was used for the statistical analysis. The comparison between allergen levels was done using non-parametric tests, as the results did not have a normal (Gaussian) distribution. Geometric means (mg) at a 95% confidence interval (CI) were calculated for each allergen level. the difference between the means was made using the Mann Whitney U test; the significant level was 5% (p < 0.05).

## RESULTS

The samples in each environment were analyzed at different times in central inland Brazilian cities (Uberlandia, Uberaba and Goiania); these cities have similar climates (humidity and temperature).

Two studies investigated the presence of household mites in two cities (Uberlandia and Uberaba). The first of these was done in 1998, and verified the presence of sensitizing mite allergen levels in five places within 64 households – sofas, floor of television rooms, beds, floor of bedrooms and kitchens. Elevated levels of Der f 1 were found in the beds of asthmatics (15.8 μg/g dust) and non-asthmatics (8.2 μg/g dust). Der p 1 levels were lower in the beds of these same individuals (asthmatics – 2.8 μg/g dust; non-asthmatics – 4.9 μg/g dust). The mean concentration between five samples showed that 91% of the samples in the homes of asthmatics, and 84% of the samples in the homes of non-asthmatics had Der f 1 levels over 2 μg/g, while 72% of the samples in the homes of asthmatics e 75% of the samples in the homes of non-asthmatics had Der p 1 levels higher than 2μg/g dust.^29^

In Uberaba, samples for analyzing mite allergens were taken from sofa and bed surfaces (mattresses, bedspreads and pillows) in 60 households in two separate periods (March and July). Higher levels were found in March in these samples; Der f 1 levels were 31.7 μg/g dust in beds and 8.3 μg/g dust on sofas. Der p 1 levels were 0.3 μg/g dust in both months.^30^

Another study done in Uberlandia assessed the presence of dust allergens in hotels, between June and October 2002; 98 samples from 20 hotels were analyzed. Mean concentrations were: Der f 1 – 11.3 μg/g dust, Der p 1 – 0.15 μg/g dust, Can f 1 – 0.3 μg/g dust and Fel d 1 – 0.11 μg/g dust.^31^

In Goiania, studies analyzed the surfaces of sofas and floors in five movie theaters. The mean concentrations were: Dep p 1 – 0.35 μg/g dust; Der f 1 – 6.85 μg/g dust; Can f 1 – 0.65 μg/g dust; and Fel d – 10.07 μg/g dust. The allergen Bla g 2 was not detected in any sample.^32^

Another study investigated interstate transport and taxis; the following mean allergen levels were found in buses with natural ventilation and buses with air conditioning: Der p 1 – 1.6 μg/g dust and 4.3 μg/g dust; Der f 1 – 0.8 μg/g dust and 2.4 μg/g dust; and Fel d 1 – 1.2 μg/g dust and 1.6 μg/g dust. The results in taxis were: Der p 1 – 0.9 μg/g dust; Der f 1 – 1.7 μg/g dust; and Fel d 1 – 1.6 μg/g dust. Although some means were not elevated, the percentage of air conditioned buses with sensitizing allergen levels was 82%, the percentage of naturally ventilated buses with sensitizing allergen levels was 62%, and the percentage of taxis with sensitizing allergen levels was 65%.^33^

An analysis of samples taken from the seats of passenger vehicles (cars) in Uberlandia found the following mean concentrations: Der p 1 – 0.24 μg/g dust; Der f 1 – 0.29 μg/g dust; Can f 1 – 1.51 μg/g dust; and Fel d 1 – 0.42 μg/g dust.^34^ The seats of school transportation vans in the same city revealed the following mean concentrations: Der p 1 – 0.15 μg/g dust; Der f 1 – 0.26 μg/g dust; Can f 1 – 1.03 μg/g dust; and Fel d 1 – 0.37 μg/g dust. ^35^

Table 1 shows the allergen level means according to each site.

**Table 1:** Mean allergen levels according to the site.

Study	Site	Mean allergen concentration (μg/g dust)			
		Der p 1	Der f 1	Can f 1	Fel d 1
Sopelete et al. (2000)	House	0,2 a 3	4,5 a 17,1	–	–
Terra et al. (2004)	House	0,30	8,3 a 31,7	–	–
Silva et al. (2005)	Movie theaters	0,35	6,85	0,65	0,07
Simplício et al. (2006)	Hotels	11,3	0,15	0,30	0,11
Pereira et al. (2004)	Buses and taxis	1,6 a 4,3	0,8 a 2,4		1,2 a 1,5
		0,9	1,7		1,6
Justino et al. (2005)	Passenger cars	0,24	0,29	1,51	0,42
Justino et al. (2005)	School transport	0,15	0,26	1,03	0,37

## DISCUSSION

The association between allergen exposure, sensitization and allergic diseases has been known since the beginning of the past century. Immunological mechanisms by which allergens cause genetically predisposed individuals to produce allergen-specific IgE and the resulting symptoms – as well as interrelated factors in allergic disease – have been studied in detail in the last two decades. The aim has been to understand the major increased in the prevalence of allergic diseases such as asthma, allergic rhinitis and atopic dermatitis in the past 30 years.

There are two main types of studies for investigating allergenic exposure: (1) those in which individuals move to different, allergen-free environments, and (2) those in which measures to reduce allergen exposure within households are undertaken.

An elegant study in the 1980s, in which asthmatic patients were admitted to hospital for rigorous and prolonged control of allergen exposure – where demonstrably local mite allergen levels were decreased – revealed that bronchial hyperreactivity decreased in these patients.^25^ Other similar studies assessed changes of environment in which patients were moved to high altitude places where mite levels were low; in these cases, sensitized patients also improved.^26^, ^27^, ^28^ A recent prospective study analyzed environment care measures for children at risk since birth, and demonstrated that there was no difference in sensitization to air allergens between the cared group and controls (in which no environmental measures were undertaken); there was, however, a difference in lung function between groups at age 3 years.^38^

Studies that aimed to decrease allergen exposure in households reached conflicting results. These studies used fundamentally different methods, such as the types of environmental control measures, the sample population, and the form by which results were analyzed.^12^, ^13^, ^14^, ^15^, ^16^, ^17^, ^18^, ^19^, ^20^ Most of the studies that showed no difference in the occurrence of symptoms of asthma and allergic rhinitis following environmental hygiene defined environmental control as orientations to reduce household allergens by using mattress covers and other point modifications.

Allergen sensitization depends on individual genetics, the amount of allergens and the duration of exposure. Certain pollens cause allergen sensitization during a relatively short pollination period (less than two week a year). Thus, it may be possible for genetically predisposed individuals – especially in childhood – to become sensitized by a few repetitive exposure periods in sites with elevated mite or animal dander allergen levels.^11^

The studies that were reviewed in this paper showed that allergens were present in a variety of environments, such as movie theaters, hotels, buses, schoolchildren transport vans, taxis and private vehicles, as well a elevated mite allergen levels in various sites within households. These studies corroborate the association between allergen exposure and sensitization by showing that there were enough allergens to cause sensitization in a variety of commonly used places. These data are similar to those in other studies of allergen exposure that have demonstrated high air allergen levels in nurseries and school, offices and files, hospitals, movie theaters and public transportation vehicles.^39^, ^40^, ^41^, ^42^, ^43^

Environmental control (tertiary preventive measures) to reduce household air allergens remains a forma recommendation for patients with allergic conditions in which allergen sensitization has been demonstrated. Secondary prevention, which involves environmental control measures done since birth in households of asymptomatic children whose parents have been diagnosed with allergic diseases, is also recommended. These individuals also require vehicle care against allergens. Individuals live in society – not only within their houses – where clinically relevant allergens may be found in places such as schools, workplaces, leisure areas and public transport vehicles. Thus, patients have to not only understand the disease itself, but also the ample possibilities of allergen exposure. Environmental control, therefore, although not sufficient to eliminate the symptoms, should become part of a wider strategy for the treatment of allergic patients and families. It would be wrong to state that environmental control in households by itself does not work in preventing symptoms in allergic patients. Failure of the environmental control measures may occur because it is rare for individuals to be completely free from contact with significant allergen levels, given the diversity of allergen exposure to which every one is subject daily.
